# Potential conflict of interest of SAGES annual meeting presentations: can we do better?

**DOI:** 10.1007/s00464-025-12487-6

**Published:** 2026-01-05

**Authors:** Brij R. Chhabra, Atul K. Madan, David S. Tichansky

**Affiliations:** 1 Southern California Bariatrics, Los Angeles, CA USA; 2 Department of Surgery, David Geffen School of Medicine at UCLA, Los Angeles, CA USA

**Keywords:** Conflict of interest, Disclosure, Industry relationships

## Abstract

**Background:**

ACCME defines ineligible companies as “those whose primary business is producing, marketing, selling, re-selling, or distributing healthcare products…”. Relationships with ineligible companies may create a potential conflict of interest (pCOI). Disclosure of pCOI is essential for integrity of educational programming and ACCME mandates disclosing these relationships. We investigated significant pCOI (spCOI), defined as total payments > $10,000 over the 2 years prior a meeting. We hypothesize that while most authors disclose pCOI appropriately at SAGES, simple disclosure of pCOI does not fully inform audiences of pCOI at scientific meetings.

**Methods:**

Payment information for authors at the 2023 and 2024 SAGES Annual Meeting was extracted from the OpenPayments™ database(https://openpaymentsdata.cms.gov). Authors not found were searched via Google, LinkedIn, affiliation websites, NPI, and/or state medical board websites. Authors were divided as Disclosed pCOI vs. Not Disclosed pCOI, the latter including those with no mention of COI, stated no relevant COI, or stated no COI.

**Results:**

Total authors were similar in 2023 and 2024(792 vs. 796). Trainees, non-physicians, and non-US physicians were excluded leaving 296 eligible authors in 2023 and 387 in 2024. The proportion of Disclosed was similar in 2023 and 2024(16% vs. 21%; *p *= ns). Presence of spCOI was similar in 2023 and 2024(25% vs 24%). Average spCOI payment was greater than $100,000 and  not statistically different between groups or by meeting year. Only 51.5% of spCOI were disclosed. The highest paid author received over $868,000 in 2023 and over $1.35 million in 2024.

**Conclusions:**

The majority of authors disclosed their pCOI at SAGES in 2023 and 2024. Undisclosed authors may still have spCOI. Disclosure allows audiences to decide if relationships impact data presented. Disclosing the mere existence of pCOI seems insufficient as the amount of dollars is substantially high for some authors. All presentations should include the dollar amounts for full disclosure of pCOI for future programs.

After SAGES made tremendous progress in increasing disclosure of potential conflict of interest (pCOI) [1–3], our group previously examined disclosure compliance in correlation to the OpenPayments database (OPD) at the 2023 SAGES meeting [4]. Finding that most authors and presenters are compliant with disclosure policies, our attention was drawn towards the magnitude of undisclosed significant pCOI (spCOI)  greater than $10,000. This led to consideration if the current disclosure policies have stalled progress and achieved a new steady state of compliance and whether we can do better.

The challenge remains that when a human brain realizes a gain, subconscious biases are created potentially without the conscious mind realizing it. [5] Unfortunately, a human loses objectivity and is prone to introduce bias without being conscious of it, which affects judgment and decisions [6]. Behavioral science recognizes that these biases are near impossible for a human being to avoid. The question of whether the magnitude of the gain correlates to the magnitude of the bias remains unanswered. However, it has been demonstrated that scientists can make adjustments in experimental design, either consciously or subconsciously, in order to increase the probability that the results will support her prior beliefs. [7] These changes can easily also align with the concept of funding bias [8].

In competition with the theory of unbiased scientific inquiry is the fact that medicine-affiliated industry continues to partner with providers, sometimes for significant fees. The influence of industry money in trials is substantial. A recent assessment of 600 well-cited studies from 2019–2022 noted that more than two-thirds (68.2%) had industry funding and half were only industry-funded [9] Further, 59.0% had industry-based authors, 46.6% utilized industry-based analysts, and 89.0% of the industry-funded trials resulted in a favorable outcome for the sponsoring company [9].

At some point in the continuum of funding, researchers are considered by industry to be unofficial employees of industry [10]. According to the United States Census Bureau, the median household income was $80,610 in 2023 [11]. The combination of these facts raises the question of how much aggregated consulting fees and speaker honorariums constitute an inflection point where an investigator should either disclose themselves as an industry employee or disqualify themselves from presenting unbiased science? If a side gig generates a whole multiple of the U.S. median household income, is that enough? This investigation takes an additional look at the compliance and degree of disclosed and undisclosed pCOI  at the SAGES meeting in 2024 as a comparator to our 2023 data [4] using the OPD as the primary source of fact to determine if progress towards complete disclosure has stopped. If so, new ideas need to be implemented to do better towards giving recipients of scientific data a clear picture of pCOI in the data creation and presentation.

## Materials and methods

This cross-sectional investigation chose to include authors who gave oral presentations at the SAGES 2023 and 2024 annual meetings. We included only plenary and scientific sessions. Our exclusion criteria included presentations that were “quick shots”, video presentations, resident/fellow forum presentations, and non-CME presentations. All of the authors of each presentation were compiled, including names, organizations and locations to help ensure that correct identification. Using these data, the CMS OpenPaymentsData database (https://openpaymentsdata.cms.gov) was initially searched. The OPD database includes physicians from the United States; thus, all non-US physicians were excluded by necessity. Since students, research assistants, residents, fellows, and other non-physician researchers were not included in the database, they were not included in this study. The total amount of payment given to each physician was recorded for the prior last two years from the year of the presentation. We arbitrarily defined spCOI as any physician receiving greater than $10,000 over the prior two-year period as we have done before.

All presentations were reviewed to determine if each author had disclosed pCOI. Each presenter was told that their second slide must declare disclosures. Every author that was included in each presentation was included in this investigation. We divided the authors into two groups: “Disclosed” and “Not Disclosed”. The Disclosed group disclosed at least one pCOI. The Not Disclosed group consisted of authors that did not disclose a pCOI. Additionally, if the authors stated they had no “relevant” pCOI disclosures or did not mention disclosures in their presentations, they were considered to be in the latter group.

Certain authors could not be found solely on the basis of their names and locations due to various reasons. For those authors, we first implemented a simple Google search to ascertain their correct name. Additionally, Linkedin website, Doximity website, practice/institution affiliation, and state medical board websites were searched to determine correct names and/or locations. National Provider Identifier (NPI) numbers were also searched for authors who had common names and/or incomplete official names in the presentations. These NPI numbers were inputted into the OpenPaymentData website and NPI was only used if it correlated with the name on the presentation.

The endpoint of this specific study was compliance with disclosure at specific meetings. All three authors were extensively involved in the data mining and processing for this project. While the scope of the project was seemingly simple, it was actually quite labor intensive to extract these data. Data were analyzed with Chi squared and two-tailed Mann Whitney tests using https://astatsa.com/ statistical program. Institutional review board exemption was received for this investigation.

## Results

**Fig. 1:**
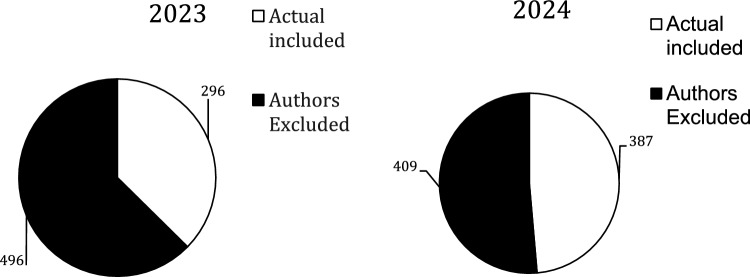
The figure demonstrates the number of included versus excluded authors by year. The numbers were similar in both years

**Fig. 2:**
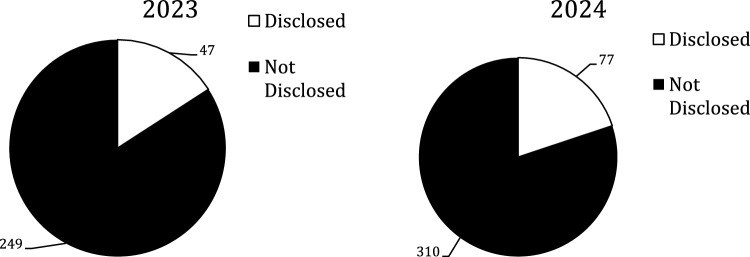
The figure shows that many of the authors stated they had nothing to disclosure for their presentation. There was no differences between 2023 and 2024

**Fig. 3:**
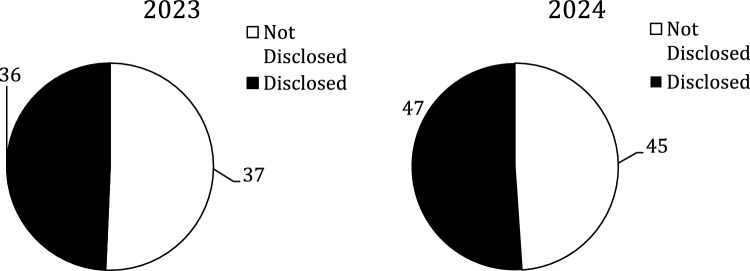
The figure shows the distribution of author with spCOI by disclosure group. No differences were seen between 2023 and 2024

**Fig. 4:**
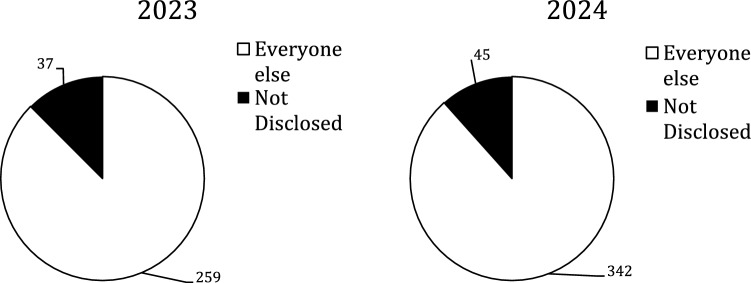
The figure shows that only about 12% of the total authors said they had nothing to disclosure but actually had spCOI. No difference was noted by years

**Fig. 5:**
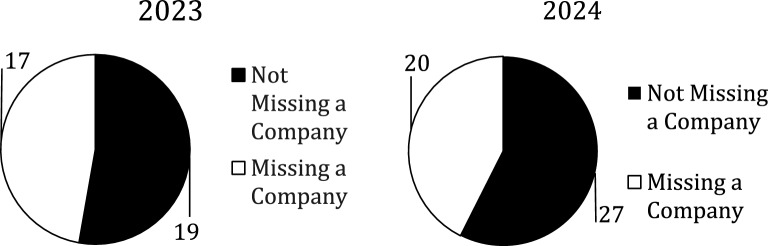
Authors who disclosed spCOI who did not list all the companies from whom they received payment. Again, no difference was noted between years 2023 and 2024

There were 1588 authors but only 683 (43%) were included; the rest were excluded due the issues discussed above. Figure 1 shows the amount that were excluded versus included by year. There were 559 (82%) authors in the Not Disclosed group and 124 (18%) in the Disclosed group; Fig. 2 shows there was no statistically significant difference between the two years. The total number of authors with spCOI in the Disclosed group was 83 vs.82 in the Not Disclosed group and the difference by year can be seen in Fig. 3. These authors with spCOI in the Not Disclosed group comprised only 12% (82/683) of total authors with no difference from year to year as seen in Fig. 4. Interestingly, 40/683 (5.9%) disclosed that they had pCOI but did not reach our definition of spCOI. Most authors (88%) did appropriately disclose their spCOI.There were 37/85 (45%) of authors in the Disclosed group that had significant potential COI and also had a payment from a company noted on the OPD but not seen on their disclosure side. Figure 5 shows the differences by year.

**Fig. 6:**
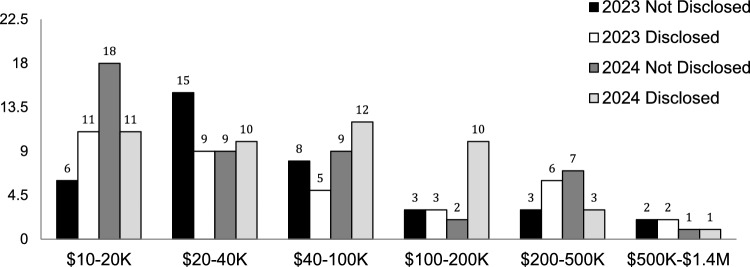
This figure shows the number of authors grouped vs total payment. While there were more authors who received lower payments, there are still a substantial number of authors who received greater than $100,000

The average payment for those who had spCOI was greater than $100,000 and did not statistically differ by year nor by group (Table 1). The median payment for spCOI was $37,500. Figure 6 demonstrates the distribution of the amount of spCOI by Disclosed vs. Not Disclosed authors as well as by year. Over a quarter of the authors (25.9%) that had spCOI received over $100,000. The highest paid author received over $868,000 in 2023 and over $1.35 million in 2024.

**Table 1:** Average total payments to authors by year and group

	2023	2024
Not disclosed	$93,056	$101,378
Disclosed	$116,447	$100,857

## Discussion

Our data show that most authors are accurate and forthcoming in their disclosures of their pCOI. However, a small proportion of authors do not disclose their spCOI. Unfortunately, the ones that did not disclose did have a large quantity in payments. While most authors did disclose appropriately, approximately half of the authors that had spCOI did not disclose appropriately. More concerning was that almost half of the authors who disclosed their spCOI did not list all their companies on their disclosure slide. Of note, a goal of this study is not to suggest that organizations verify what is reported. The process of compiling data for this study would essentially be the same process for the organization if verification was desired. This is an extraordinarily labor-intensive process for a society, whereas the alternative is that the authors do it correctly and comprehensively.

A strength of this study is that the data is all publicly available information. Our study does have some limitations.

First, we could not truly know the true pCOI. We do not have access to author’s financial records nor did we ever want to. By definition, all presenters have a pCOI. They have an interest to make sure that they have interesting data to present. The self-serving interest will always exist and is hard to quantify. The other weakness is that we do not know the accuracy of the OPD dataset. The OPD is somewhat self-reported and time-delayed data and may understate the existing relationships. Even if we were able to find the correct author, the persons inputting the data may not have been so thorough. While physicians are allowed to challenge the data seen on OPD, most of us do not. One of the authors of the paper (AKM) has payment information that is probably from his namesake. That information was never corrected. Despite this, the data we looked at seems to be in line with our specialty and the industry that we deal with.

We did note that some authors stated no “relevant” disclosures. Societies should not allow such terminology. It should allow the audience to determine relevance. If the authors truly have no relevant disclosures, we feel that our audience has the whereabouts to come to that conclusion. Adding a qualifier such as “relevant” tends to allow ambiguity that contradicts the spirit of full disclosure.

One interesting observation that we noted was that there were more than a few authors that had an exorbitant amount of money from industry. This becomes an issue since ACCME guidelines do not allow for employees of eligible companies to participate in CME activities. If an author is getting paid greater than five times the U.S. Median household income, the line between consultant and employee blurs. It can be interpreted that these authors may actually be ineligible to present at CME activities. At very least, their audiences should know the degree to which conflict of interest could occur. The revelation of this investigation is that the amount payments received by authors is extremely important to be disclosed. It can seem disingenuous to suggest that an author who gets paid $500,000 should have the same disclosures slide as an author who only gets paid $500. And more importantly, the potential bias would not be the same between these two authors.

The question is what level, if any, should be set before an author is considered a de facto employee of the company. We suggest two times the U.S. median household income per year ($161,220 for 2023) from any one specific company. In those cases, consideration may be a cutoff to declare them as a “de facto employee”/ “paid contractor” and mentioned as such in the program and in the abstract. This may be best implemented during the submission of the abstract itself. This is a novel concept in terms of disclosure but we feel that these data show that it may be necessary. Some may see this as excessive; it is not. Although rare, practicing surgeons do at times become actual employees of industry. The assumption is that those who already get paid a significant amount from industry are more likely to become these employees. A potential pre-employment  relationship is another reason why disclosure of pCOI is so important for any presentation.

Another suggestion on how we can do better regarding disclosure of pCOI  is to provide more sophisticated instructions for presenters. SAGES already does an excellent job in explaining what and how to disclosure to committee members. They have an infographic that helps members understand what a disclosure is. It is imperative that all authors are given this information to review and provide accurate data for the disclosure slides of the presentations. The instructions can be given at the same time the general instructions are given to the presenter. Additionally, moderators should challenge the presenters if they do not give disclosures. Ideally, a system  could be created that the data from disclosures are automatically created from the OPD website and those should be given while the presenter is introduced. These data should include the author’s name, source and amount of money, and the reason behind the payment.

One issue we found during our investigation is that some doctors were not found in the OPD dataset. While occasionally some doctors had no payments received, often exclusion from the OPD is for other reasons. To mitigate this, correct official names should be required in all presentations. The correct official name would be the name that is associated with their correct NPI number. This would correct name changes due to marriage or divorce, as well as authors known by their nickname or shortened names. Additionally, this would correct for the doctors who use their middle name or even a “westernized” version of their name. We should require the actual NPI name as well as the lived name of the presenters be available.

While this investigation focuses more on research presentations, all surgical societies need to be aware of spCOI of large amounts elsewhere. For that reason, all committee chairs, executive council members, and committee members should also disclose not just the existence of a pCOI but also the amount of payments received. As a society, SAGES can set the example of full transparency. While industry is a great partner, we need to make sure that industry money does not have undue influence on our society. We will never have a society that is devoid of potential conflicts. Some of the most prolific authors and forward thinkers are purposely sought out by industry as they should be. However, we do not want “de facto” employees of industry making major decisions for our society. We must hold ourselves to a higher standard because much of research has a direct impact on patient care.
